# Supplementary material for: miR-152-3p Represses the Proliferation of the Thymic Epithelial Cells by Targeting Smad2
Source: Genes (Basel). 2022 Mar 24;13(4):576. doi: 10.3390/genes13040576 (PMC9028272; doi:10.3390/genes13040576)
Supplement: Supplementary file 1 [file genes-13-00576-s001.zip › Supplementary Figures.pdf]

Supplementary Figure 1

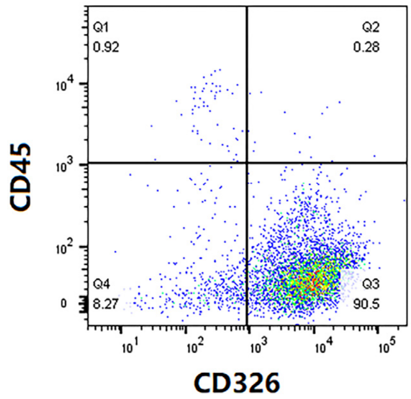

Figure S1. The purity of enriched TECs cells was detected by flow cytometry.

Supplementary Figure 2

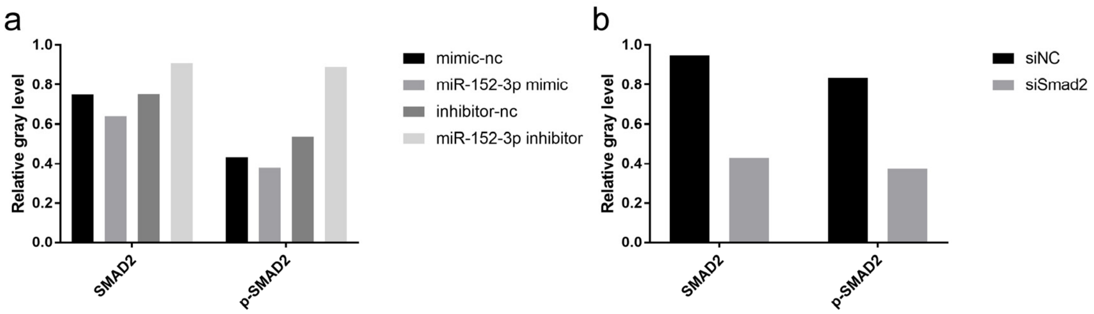

Figure S2. Grayscale analysis of WB bands. (a) miR-152-3p affects the expression of SMAD2 protein in MTEC1 cells. (b) Silencing Smad2 decreased the expression of SMAD2 protein. Relative gray level = target band/β-actin.
